# Bibliometric, network, and thematic mapping analyses of metaphor and discourse in COVID-19 publications from 2020 to 2022

**DOI:** 10.3389/fpsyg.2022.1062943

**Published:** 2023-01-16

**Authors:** Reem Alkhammash

**Affiliations:** English Department, University College-Taraba, Taif University, Taif, Saudi Arabia

**Keywords:** bibliometric analysis, co-citation analysis, thematic mapping analysis, metaphor and discourse, COVID-19, VOSviewer

## Abstract

The research contributions of metaphor as part of (critical) discourse studies have flourished during COVID-19; hence, it is necessary to consider their progress and foresee their future growth. To obtain a comprehensive understanding of COVID metaphor research in discourse and to identify the most recent research foci, bibliometric, network, thematic mapping and word cloud analyses were conducted in this study. The results showed that (1) research on COVID metaphors is largely shaped by Critical Discourse Analysis research approaches and methodologies; (2) the research production has investigated traditional genres such as news and emerging genres, including social media and multimodal data; and (3) research highlights the role played by metaphors in persuasion in public discourse. The findings of this study can assist future research in this or related fields by providing an overview of metaphor research in crisis communication.

## 1. Introduction

The COVID-19 pandemic prompted changes in the social order and economic and business ties, in addition to a substantial increase in social fear, tensions, and skepticism. It has inevitably manifested in language use and discursive practices, which largely rely on discourse. Previous studies have emphasized the metaphor's effectiveness in fostering a sense of urgency in healthcare organizations reacting to the pandemic (e.g., Jetly et al., 2020; Maxwell et al., 2020). Other research discussed which metaphors have been used for the pandemic, how metaphors are used and the implication of some metaphorical usage (see Chapman and Miller, 2020; Semino, 2021; Musolff, 2022). Given the increasing number of international publications on COVID-19-related metaphors and discourse research, the synthesis of the growing research output is a profound step toward advancing the role played by language in health communication and promotion (see Semino, 2021). Bibliometric analysis is a recent method used to examine the trends in scientific production of a specific field (Wang et al., 2022). Bibliometric analysis uses statistical and scientific mapping approaches to quantify research contributions. Linguists have used bibliometrics since its inception to examine the influence of scientific literature and research trends in particular branches of linguistics, such as translation research (Van Doorslaer and Gambier, 2015; Zanettin et al., 2015), applied linguistics (De Bot, 2015; Lei and Liu, 2019a), corpus linguistics (Lei and Liao, 2017), fuzzy linguistic research (Chen et al., 2019), and second language (Lei and Liu, 2019b).

More recently, bibliometric analyses of discourse research have emerged in major discourse analysis journals such as Discourse and Society and Discourse and Communication (Huan and Guan, 2020; Xiao and Li, 2021). Casting a wider net in the field of critical discourse analysis, Xiao and Li (2021) conducted a bibliometric and co-citation study of critical discourse studies that were published in the Web of Science (WoS) database between 2011 and 2020 to unravel research trends. In the retrieved 8,137 studies, they discovered information about the scope of research, collaboration between countries and institutions, most prolific authors, and emerging research trends. They found that discourse studies extended to research in linguistics, communication, education, business, economics, and social issues. Collaborative research peaked in the United States and Europe. Furthermore, David Machin was the most prolific author, and TA Van Dijk was the most cited author. Discourse and Society had the most CDA/CDS-related articles. CDA research is influenced by theories and techniques from cognitive linguistics, corpus linguistics, and multimodal discourse analysis.

To the best of our knowledge, this is the first study to examine the scholarly contribution of COVID-19-related metaphors and discourse from bibliometric, network, and thematic mapping perspectives. This is particularly timely considering Peng and Hu (2022)'s recommendation of the need to investigate COVID-19 discourse especially the applications of Conceptual Metaphor Theory. A total of 327 valid research papers and book chapters were retrieved using WoS and Scopus. By collecting and evaluating COVID-19 metaphors and discourse-related articles, this study aimed to provide a direction for metaphor experts. In essence, the following research questions (RQs) were addressed:

**RQ1:** Which authors, journals, and countries are the most prolific in COVID-19 metaphors in discourse research?
**RQ2:** What are the most cited authors, journals, and references in metaphor research of COVID-19 discourse?
**RQ3:** What are the current themes and frontiers in the direction of COVID-19 metaphor based on discourse research?

## 2. Literature review

### 2.1. Metaphor and COVID-19 discourse studies

According to conceptual metaphor theory (CMT) (Lakoff and Johnson, 1980), people systematically structure the ABSTRACT (for example, PANDEMIC) in terms of the CONCRETE (e.g., WAR). As the source domain is more tangible and perceivable, it embodies knowledge that people can draw on to conceptualize more abstract notions. For example, in the source domain JOURNEY, everyone has tangible experiences related to embarking on journeys used to map onto ACHIEVING GOALS (Kromhout and Forceville, 2013).

The vast majority of studies that combined discourse and cognitive metaphor theory have highlighted metaphor's persuasive and evaluative potential (e.g., Maalej, 2007; Ferrari, 2018; Charteris-Black, 2019). However, the specifics of how people can use metaphor to persuade and evaluate has rarely been investigated. According to Deignan (2010), a metaphor can communicate an evaluation (positive or negative) through four different mechanisms: creating entailments, using scenarios (Musolff, 2016), deliberately selecting source frames that are meaningful to specific demographics, and exploiting the connotations of literal meanings.

Discourse studies on COVID-19 have used corpus linguistics as a primary method of analysis. Corpus linguistics studies how language use mirrors the COVID-19 outbreak and how attitudes and beliefs are conveyed through language use (Mahlberg and Brookes, 2021, p. 441). Scholars have gathered corpora to explore the pandemic from a linguistic perspective. The Coronavirus Corpus (Davies, 2021) includes COVID-related news stories with at least two occurrences of coronavirus, COVID or COVID-19, or phrases such as “at-risk,” “cases,” “confirmed,” “contagious,” “hydroxychloroquine,” “outbreak,” “pandemic,” and “stay-home.” Corpora helps us understand pandemic language. COVID-related corpora contain texts and multimodal data such as memes, political cartoons, and health messages (see Abdel-Raheem, 2021; Dynel, 2021; Sarfo-Kantankah et al., 2021).

Regarding COVID metaphors, previous research has noted the predominant use of the WAR metaphor by public figures to describe efforts to minimize the spread of COVID-19 in the political and news discourses (see Bates, 2020; Chapman and Miller, 2020; Pfrimer and Barbosa, 2020; Isaacs and Priesz, 2021; Semino, 2021; Hanne, 2022). WAR metaphors can motivate the public's support for political action. For example, the UK government described COVID-19 as a WAR (and *a battle with many fronts*), and the former US Vice President Mike Pence described American's effort to eliminate the virus as winning a WAR (we are *winning the fight against the invisible enemy*). In the WAR metaphor, POLITIZATIONS are described as GENERALS, DOCTORS, or SCIENTISTS; PUBLIC SERVICE WORKERS are described as SOLDIERS; COUNTRIES are metaphorized as a BATTLEFIELD; MEDICINE is metaphorized as a WEAPON; the HUMAN BODY is described as a FORTRESS; and MEDICAL SOLUTION is described as WAR STRATEGIES, and so on Bates (2020). Many linguists have resisted using the WAR metaphor to conceptualize COVID-19 and have called for alternative uses of metaphors such as the FIRE metaphor (see, for example, Semino, 2021).

However, in the medical and political cartoon discourses, the PERSON metaphor is widely used to describe the pandemic. Personifying diseases in discourse facilitates our understanding of the world and our reaction to it. Hence, in medical discourse, COVID-19 has been personified as an INTRUDER, SPY, STRANGER, or GUEST. For example, the personification of COVID-19 as a resident in the human body (humans did indeed *welcome the virus* in—*to our habitats, our houses, and our noses*) demonstrates this point (Heffernan, 2020). Another PERSON metaphor is evidenced in the research by Abdel-Raheem (2022). Abdel-Raheem analyzed political cartoons about COVID-19 and revealed the use of gendered metaphors such as the male personification of the coronavirus as a SPORTSMAN, a POLICEMAN, a PRESIDENT, a MAILMAN, a WAITER, a MALE BOXER, or an ARM-WRESTLER and the female personification of the virus as a BELLY-DANCER.

### 2.2. Bibliometric analysis in discourse studies

Recently, two studies in discourse analysis have focused on the genre of news and have combined bibliometric analysis with other techniques of analysis, such as co-citation analysis (Mu and Ma, 2022; Wang et al., 2022). Wang et al. (2022) used citation analysis to provide a deep understanding of the news in discourse studies by examining 606 articles retrieved from the WoS database from 1994 to 2021. They identified influential researchers, institutions, and countries. Most of the studies cited journals and references and emerging research frameworks and methods. In addition, western authors were the most prolific and cited authors. However, since 2002, authors from developing countries such as China, Malaysia, South Africa, and Indonesia have become influential. Communication and linguistics and language theories, approaches, and methods, such as systematic functional grammar, the appraisal framework, discursive news value, multimodality analysis, corpus linguistics, CDA, and content analysis, were frequently used in this field.

In a CiteSpace-based analysis of CDA studies of news, Mu and Ma (2022) found that Ljubljana University topped the research production in news discourse analysis. The University of Ljubljana in Slovenia, Lancaster University in the UK, and Hong Kong Polytech University in China collaborated on research projects. Paul Baker, Christopher Hart, David Machin, and Michal Krzyanowski generated a highly influential articles, guiding research in this field. Norman Fairclough was ranked first in co-citations, and Discourse and Society, edited by Van Dijk, had the most news-related CDA articles. CDA in news discourse includes applications of theories and methods from corpus discourse analysis, multimodal discourse analysis, systematic functional grammar, conversational analysis, and content analysis.

In metaphor and discourse, research is mostly restricted to a limited description of synthesis of previous literature, and bibliometric analysis remains in early inception. Despite being limited to metaphor research of COVID-19 metaphors in 2020, Silva (2020) conducted an analysis of research production and found that the conceptual metaphor COVID-19 AS AN ENEMY was widely used in news discourse during the pandemic. Abdul Malik et al. (2022) provided an overview study of metaphor research and closely examined 23 studies between 2015 and 2020. They found five major research trends in metaphor research: *conceptualizations and patterns of metaphors, metaphor and health, metaphor, ideology, and persuasion, metaphor and culture*, and *metaphor and languages* (Abdul Malik et al., 2022). Other content analyses were used such as the distribution of journals over the years, the corpus tools that were used in previous studies, the general corpora used, and whether previous studies employed theoretical frameworks such as conceptual metaphor theory.

## 3. Data and methodology

**Figure 1:**
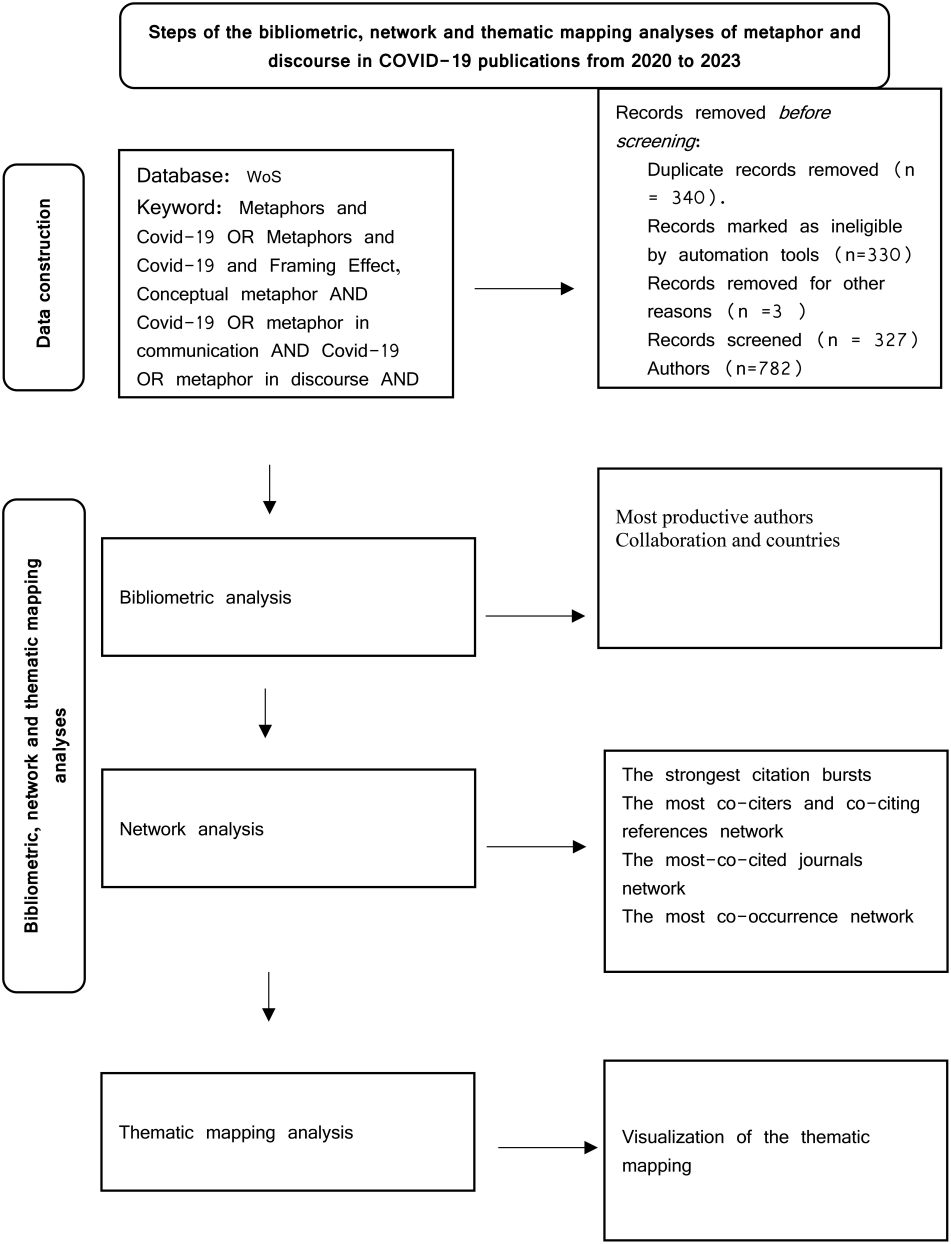
Study flowchart.

Regarding the data collection and methods of the analysis stage, the study was divided into four sub-stages (see Figure 1).

### 3.1. Data

WoS and Scopus were used to amass a large collection of international literature on metaphor and discourse in the COVID-19 pandemic. To ensure the systematic nature of this study, a combined search on WoS and Scopus databases was conducted using a set of search terms: metaphor AND COVID-19 AND discourse and conceptual metaphor AND COVID-19 discourse in the title or abstract. The search results yielded hundreds of articles, dissertations, conference papers, and books. Once the retrieval was completed, the relevant articles were checked thoroughly to determine whether the article could cover the main results of Metaphor in COVID-19 discourse. Keywords, headings, and abstracts were inspected to identify relevant research. Web of Science (WoS) and Scopus retrieved 327 relevant articles from 2020 to 2022 with the following keywords: metaphors AND Covid-19 OR metaphors AND Covid-19 AND framing OR conceptual AND metaphor AND Covid-19 OR metaphor AND communication AND Covid-19 OR metaphor AND discourse AND Covid-19. This was followed by extracting and preserving important information from these papers (e.g., titles, authors, author institutions, source journals, abstracts, keywords, and references) as a csv file for conducting the analyses. The retrieval time of these articles was August 30, 2022. The records retrieved from WoS and Scopus contained extensive, detailed information regarding authors, publication years, universities, and the references cited. This study adopted steps suggested by Echchakoui (2020) to merge the WoS and Scopus databases as follows:

- Converting both WoS and Scopus databases into bibliography files using Endnote.
- Converting bibliography files of the two databases into bibtex files using the Bibliometrix package (Aria and Cuccurullo, 2017).
- Unifying the field tags in both databases using Microsoft Excel.
- Merging the two databases using the Bibliometrix package.
- Removing duplicates as the result of merging using Microsoft Excel.

### 3.2. Methodology

#### 3.2.1. Bibliometric analysis

The study conducted a bibliometric analysis because its merits outweigh the selection of systematic literature review as a primary method of analysis. A limited focus of investigation is necessary to conduct a systematic literature review (SLR); for example, a metaphor and discourse study of COVID-19 that is specific to one genre such as social media or one culture/language. On the contrary, bibliometric studies handle research with a wider scope such as metaphor and discourse publications related to COVID-19 (Donthu et al., 2021). Another difference between SLR and bibliometric analysis is that SLR tends to rely on qualitative analysis, which might lead to biased results, while bibliometric analysis is quantitative in nature, thus decreasing the probability of the study results to be biased (Donthu et al., 2021).

Bibliometric analysis is premised on considering citations as an efficient and significant predictor for evaluating the impact of different publications or authors on specific fields of research (Culnan et al., 1990). Although citation behavior can be influenced by factors such as an article's ease of access or negative citations, citation totals can provide an unbiased estimate of a publication's significance (Culnan, 1986, 1987).

#### 3.2.2. Co-citation analysis

In recent years, various scientometric and co-citation analytic investigations were conducted to examine published studies' referenced works to evaluate how relevant and connected they were to the subject of research (Mu and Ma, 2022; Wang et al., 2022). Co-citation analysis is a popular method in bibliometric analyses (Acedo et al., 2006), which facilitates the investigation of scholarly links between influential research outputs in an area of a study and the mapping of the intellectual structure of the study area (Calabretta et al., 2011). In this study, research papers were collated and retrieved to look for semantic correlations in subsequent citing papers. The objective was to extract relationship patterns across target studies to advise metaphor researchers about core articles in this subject and to show the associations across research to provide recommendations and suggestions for future studies. A total of 27,032 references and 799 authors were extracted from the studies of interest to determine their semantic relationships. VOSviewer was used to cluster and display the network patterns from abstracts and keywords (Van Eck and Waltman, 2010, 2014; www.vosviewer.com). VOSviewer is a software application for building and displaying bibliometric networks. VOSviewer is used to examine a clustering analysis of publications at the aggregate level. Additionally, VOSviewer can be used to create maps of authors or journals based on co-citation data or keywords based on co-occurrence data. The program provides a viewer that permits an in-depth examination of bibliometric maps. VOSviewer can display a map in a variety of ways, each highlighting a distinct aspect of the map. It can zoom, scroll, and search, allowing for a detailed examination of a map. VOSviewer's viewing capabilities are particularly useful for maps containing at least a substantial number of features (e.g., at least 100 items). Furthermore, VOSviewer uses the VOS mapping technique to construct a map (Van Eck and Waltman, 2007a), where VOS represents the visualization of similarity (see Van Eck and Waltman, 2007b). VOSviewer can display maps created using any suitable mapping method.

#### 3.2.3. Thematic mapping analysis

In the thematic mapping analysis, the co-occurrence network clusters were shown as bubbles in a graph according to Callon's centrality and density rank (Callon et al., 1991). The cluster's word occurrences determine the bubble size. The X-axis depicts network cluster centrality, or the degree of interaction with other graph clusters, and measures the significance of a study theme. The Y-axis represents density, a metric of a cluster network's internal strength and theme growth (Cahlik, 2000; Cobo et al., 2011, 2015). By graphing themes, we found (a) motor themes (first quadrant, top right): the cluster network has high centrality and density, signifying themes are well-developed and crucial for structuring a research subject; (b) niche themes (second quadrant, top left): themes with high density and low centrality, signifying that they are of limited relevance; (c) emerging or declining themes (third quadrant, left bottom): themes with low centrality and low density, implying that they are minimally developed and marginal; (d) basic themes (fourth quadrant, right bottom): they have high centrality and low density. These themes are vital for transdisciplinary research issues. In the visual representation, identifying the trajectory is shown by dividing time into segments. In other words, a movement toward the upper right over time indicates a rising trend, whereas a path toward the lower left indicates a declining trend.

#### 3.2.4. Word cloud analysis

Word clouds visually depict word frequency. The frequency at which a term appears in the material being analyzed determines the size of the text in the image representation. Word clouds are used to find the center point of written text (Atenstaedt, 2012). In bibliometric studies, the use of a word cloud to evaluate the most prevalent words indicates that most of the work is concentrated in those areas. In addition, words in smaller letters indicate potential study directions (Mulay et al., 2020). A word cloud translates texts to tags, which are words whose relative value can be viewed in the resulting cloud through their size and color (Mulay et al., 2020).

## 4. Results

### 4.1. Most prolific authors, institutions and countries in COVID-19 metaphors

Examination of the stages of development, accumulated knowledge, and growth and development of discourse studies and metaphor in COVID-19 is facilitated by identifying the current annual trends in publishing output. Metaphor and discourse during COVID-19 research has increased from 30 studies in 2020 to 140 studies in 2021, and 157 studies in 2022, with an annual growth rate of 125%. The average citation per document is 2.7, and the average citations per year per document is 1.3. Furthermore, multidisciplinary fields are receiving a growing amount of attention, which may be a factor in the field's significant growth in 2021 and 2022.

#### 4.1.1. Most prolific authors

Table 1 lists the top six most prolific researchers from 2020 to 2023. Ahmed Abdel-Raheem dominated the list. Anaïs Augé, Andreas Mussolff, Sameer Naser Olimat, Laura Filardo-Llamas, and Elena Semino made significant contributions to the advancement of metaphor research through their notable publication accomplishments. For example, Abdel-Raheem (2021, 2022) published research on multimodality and metaphor. Additionally, Abdel-Raheem and Alkhammash (2022) conducted a novel experimental analysis of the framing effects of news and political cartoons on Saudi women's willingness to acquire the COVID-19 vaccine, which contributed to the development of experimental studies of metaphor. Semino (2021) problematized the use of WAR as a productive source domain to metaphorize COVID-19 and looked for other liberating and innovative source domains in a social media project entitled #reframecovid, which collected metaphorical examples from different languages and cultures. In addition, Musolff (2022) analyzed the use of WAR metaphors by political leaders to describe their efforts to deal with COVID-19.

**Table 1:** Most productive authors of metaphor research during COVID-19.

**No**	**Authors**	**Articles fractionalized**
1	Abdel-Raheem A	4.50
2	Augé A	2
3	Musolff A	2
4	Olimat SN	2
5	Filardo-llamas L	1.50
6	Semino E	1.20

#### 4.1.2. Most prolific journals

To become familiar with the leading journals in a particular field, one must consider the quantity of citations such as *h*-index, *g*-index, and *m*-index (see Table 2). An *h*-index is a measure of the cumulative effect and performance of a journal's scholarly output. For example, an *h*-index of 10 indicates that a journal has published a minimum of ten papers with at least ten citations each. A g-index is computed based on the number of citations received by a given journal. An *m*-index is the *h*-index divided by the duration of a journal's active career. Journals such as Social Semiotics, Journal of Language and Politics, Discourse and Communication, Discourse and Society, Discourse, Context and Media, and Journal of Pragmatics are considered the leading journals in linguistics, discourse analysis, and pragmatics, ranking among the top 10% in the category of linguistics and communication, as per the Journal Citation Report 2021. Other journals such as PloS ONE and Health Communication have a wider scope and publish multidisciplinary research. WoS has published some noteworthy new journals (ESCI) such as *Gema Online Journal of Language* and *The Russian Journal of Linguistics*.

**Table 2:** Top ten most influential journals in metaphor and discourse on COVID-19.

**Journal**	***H*-index**	***G*-index**	***M*-index**
Gema Online Journal of Language Studies	4	6	1.333
PloS ONE	3	3	1
Health Communication	2	2	1
Journal of Language and Politics	2	2	2
Russian Journal of Linguistics	2	3	0.667
Social Semiotics	2	2	1
Discourse and Communication	1	1	1
Discourse and Society	1	1	0.5
Discourse, Context and Media	1	1	0.5
Journal of Pragmatics	1	1	0.5

#### 4.1.3. Most prolific countries

**Figure 2:**
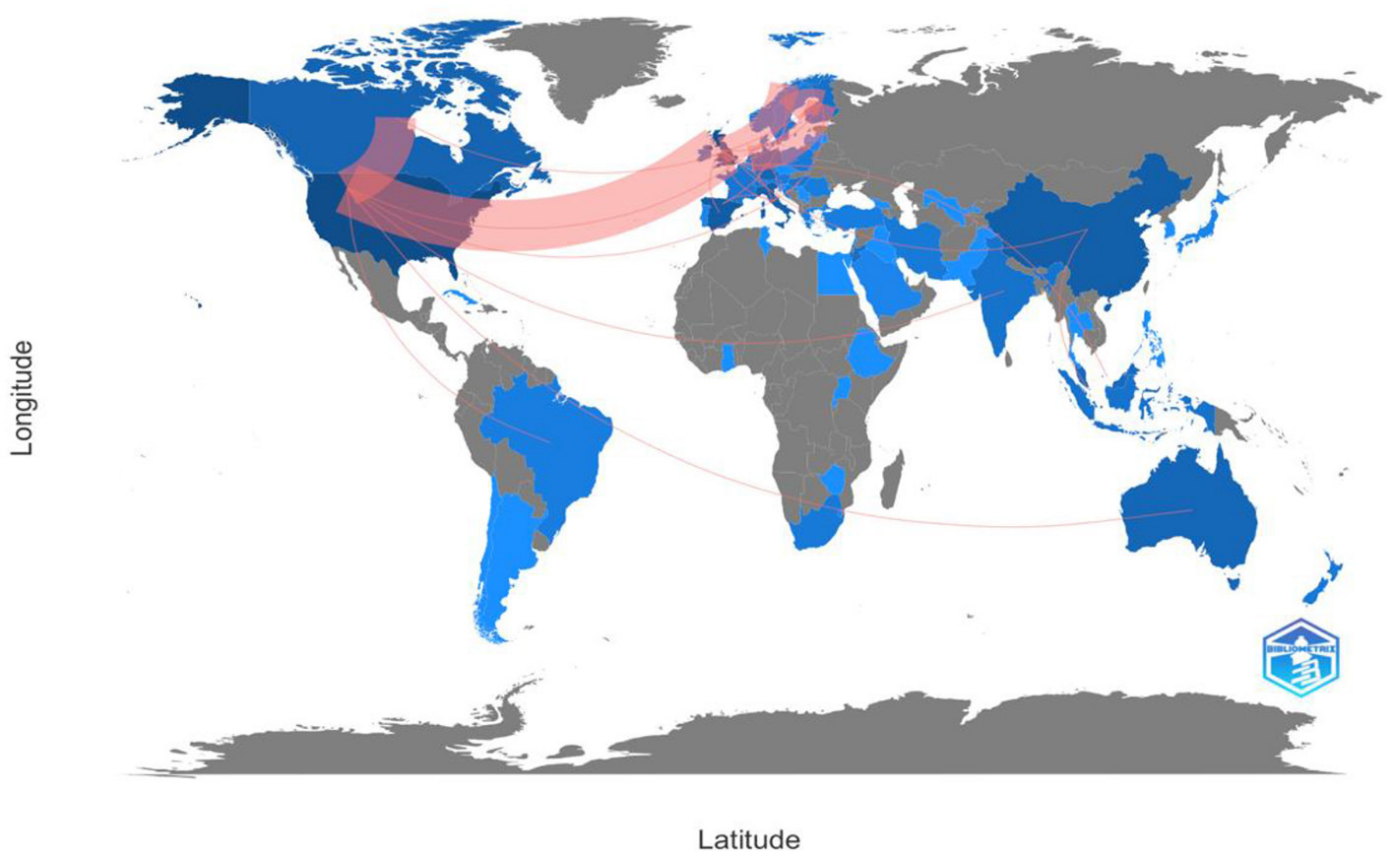
Collaboration world map of the data.

As seen in Figure 2, numerous countries have published articles on metaphor and discourse on COVID-19. However, the majority of published articles were confined to a few countries. English-speaking countries were the most well-represented. In addition, there was a significant presence of European countries. Some countries in Asia, South America, and Africa were present to a lesser extent.

The results of the collaboration were reflected in the country of production, with the United States (37 papers), the United Kingdom (32 papers), Australia (7 papers), and Canada (8 papers) representing the English-speaking countries. Other European countries contributed to the research collaboration, such as Germany, the Netherlands, and Spain, with 14, 11, and 17 papers, respectively. Other Asian, South American, and African countries contributed to the scientific production of metaphor research in discourse such as China (16 papers), Malaysia (8 papers), Brazil (2 papers), Côte d'Ivoire (1 paper), and Saudi Arabia (1 paper).

**Figure 3:**
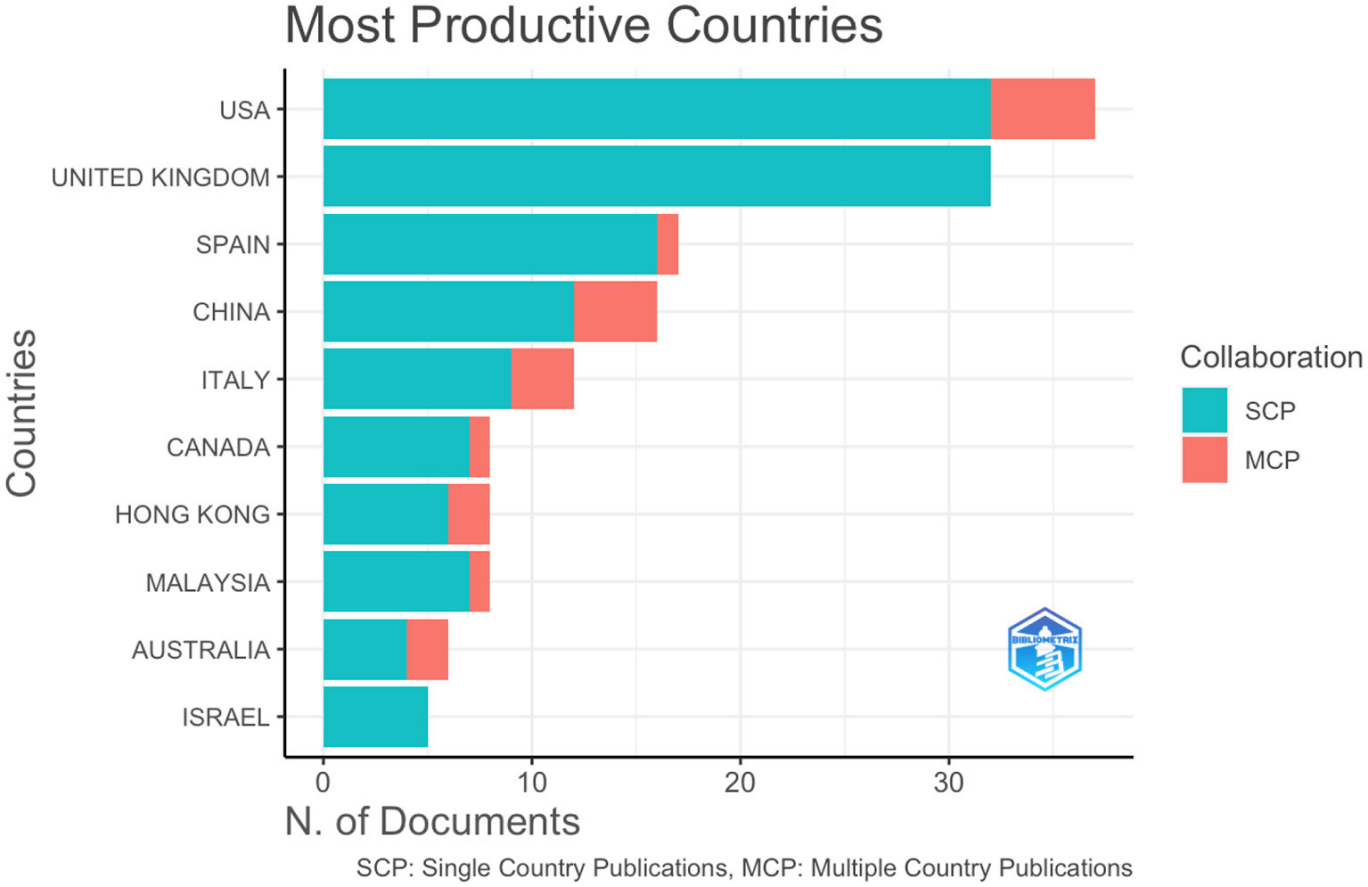
The global contribution by country of corresponding author's country.

Top ten countries with single country publications (SCP) are plotted in Figure 3, with the United States ranking the first and the United Kingdom ranking second in the most productive countries in COVID-19 metaphor and discourse research. Furthermore, United States, China, Italy, Hong Kong/ China, Australia, Spain, Canada, and Malaysia were the most productive countries with multiple country publications (MCP), while the United Kingdom and Israel had no collaboration outside their countries.

### 4.2. Most cited authors, journals and references in COVID-19 metaphors

#### 4.2.1. Most cited authors

**Figure 4:**
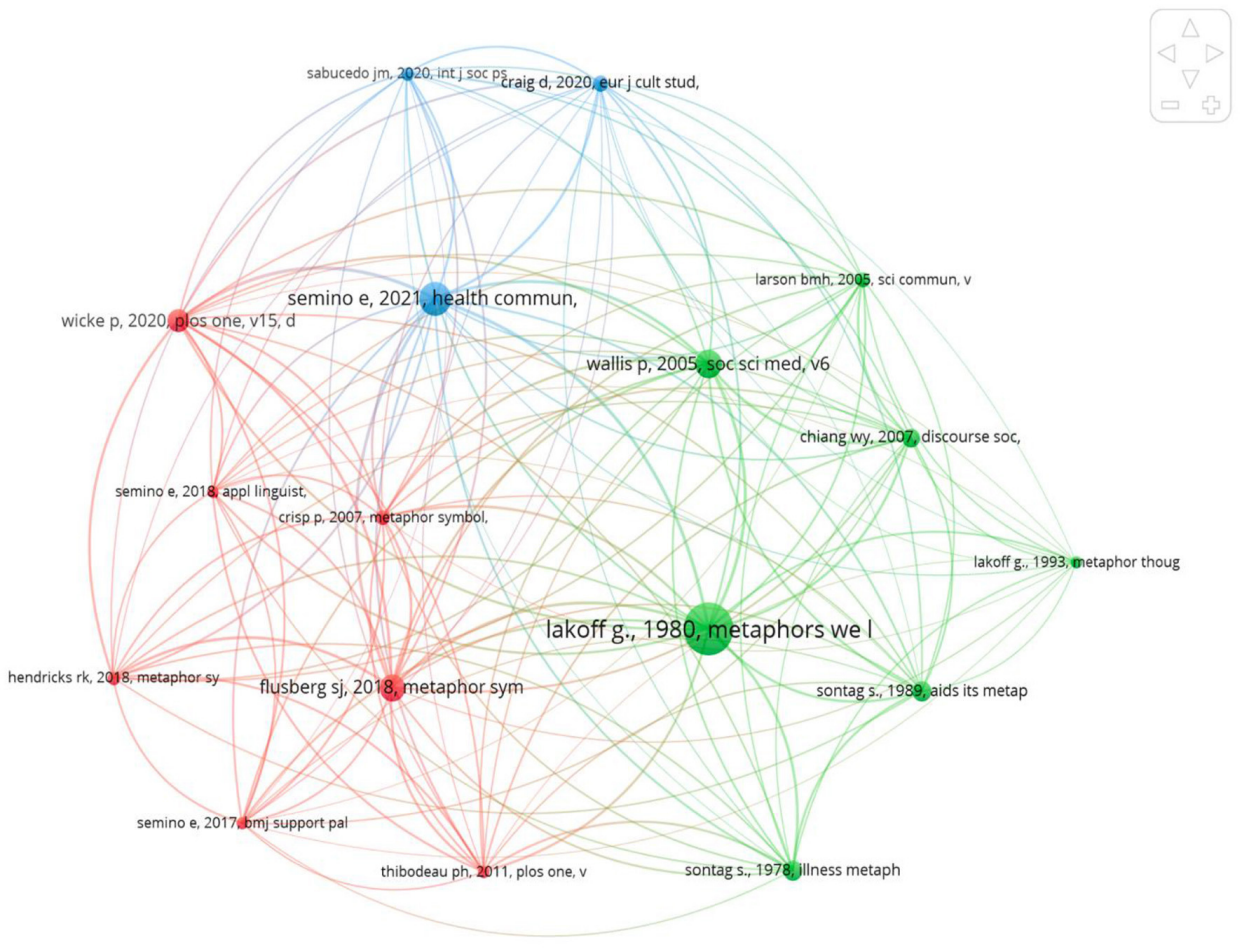
Visualization of the most frequent co-citers and co-citing references network.

Figure 4 show the most co-cited references, which share semantic functions. Three clusters were identified in Figure 4. Cluster 1 represents seminal work in metaphor theory or ground-breaking research in metaphor in health communication and consists of seven references (green color): Sontag (1979), Lakoff and Johnson (1980), Sontag (1989), Lakoff (1993), Larson et al. (2005), Wallis and Nerlich (2005), and Chiang and Duann (2007). Lakoff and Johnson (1980) and Lakoff (1993) show that in Conceptual Metaphor Theory (CMT), people map in systematic ways the abstract in terms of the concrete. They refer to the abstract as the target domain and they call the concrete the source domain. Wallis and Nerlich (2005) investigated metaphors in British media coverage to frame SARS (SARS). They found that used SARS as a source domain to describe refugees and immigrants. They found out that WAR is used as a source domain to describe the virus when the virus threatens nations. They also found out that right-wing politicians and media in the UK utilized the SARS to oppose immigration and refugees. Chiang and Duann (2007) found that SARS are framed using WAR metaphor. Sontag (1979, 1989) showed that disease news often combines invasion images and war metaphors to describe diseases. Larson et al. (2005) have examined the use of militaristic metaphors (e.g., “battle” or “war”) to emphasize the urgency of addressing invasive health threats.

Cluster 2 consists of seven references (red color): Crisp (2008), Thibodeau and Boroditsky (2011), Flusberg et al. (2018), Hendricks et al. (2018), Semino et al. (2018), and Wicke and Bolognesi (2020). Flusberg et al. (2018) showed that public discourse often uses WAR metaphors to discuss diseases like cancer. Wicke and Bolognesi (2020) analyzed COVID discourse on social media, they found that although the family frame includes a larger range of topics, WAR, a conventional figurative frame, was employed most often in social media discourse about COVID (Crisp, 2008). Thibodeau and Boroditsky (2011) provided experimental evidence that metaphors influence our conceptualization, reasoning, decision-making, and action. Hendricks et al. (2018) investigated whether metaphors impact how we judge, or appraise, an emotionally distressing circumstance like an illness. They found out that patients' reactions and assessments of their hardships can be affected by metaphors of WAR and JOURNEY. Semino et al. (2018) conducted corpus-based research of postings to an online cancer forum and found that a patient's relationship with the illness can be affected by the framing invoked using WAR metaphors.

Cluster 3 shows recent research of metaphors of the COVID-19 pandemic and contains three references (blue color): Sabucedo et al. (2020), Kalinec-Craig et al. (2020), and Semino (2021). Semino (2021) showed that COVID-19 media coverage uses WAR metaphors. Sabucedo et al. (2020) showed that WAR metaphor in the pandemic have been criticized for its lack of relevance to communicate the need to adopt self-limiting behavior such as strung at home to avoid getting COVID-19. Kalinec-Craig et al. (2020) showed the merits of using metaphorical intervention for learning. It is worth noting that Lakoff and Johnson (1980) has the most co-cited articles and is contained in the three main clusters of co-citation. The threshold number of minimum citations for a cited reference is three. Table 3 demonstrates the number of co-citations for the 17 articles.

**Table 3:** Co-citations of the co-citers and co-citing references.

	**Authors**	**No. of co-citations**	**Total links**
1	Lakoff and Johnson (1980)	50	132
2	Semino (2021)	31	122
3	Flusberg et al. (2018)	25	111
4	Wallis and Nerlich (2005)	26	101
5	Wicke and Bolognesi (2020)	21	99
6	Crisp (2008)	13	85
7	Thibodeau and Boroditsky (2011)	11	75
8	Lu and Chiang (2007)	17	74
9	Hendricks et al. (2018)	12	74
10	Sontag (1979)	18	67
11	Semino et al. (2018)	11	64
12	Sontag (1989)	18	63
13	Proctor and Larson (2005)	13	62
14	Sabucedo et al. (2020)	12	59
15	Semino et al. (2017)	11	59
16	Kalinec-Craig et al. (2020)	14	57
17	Lakoff (1993)	11	28

#### 4.2.2. Most cited journals

**Figure 5:**
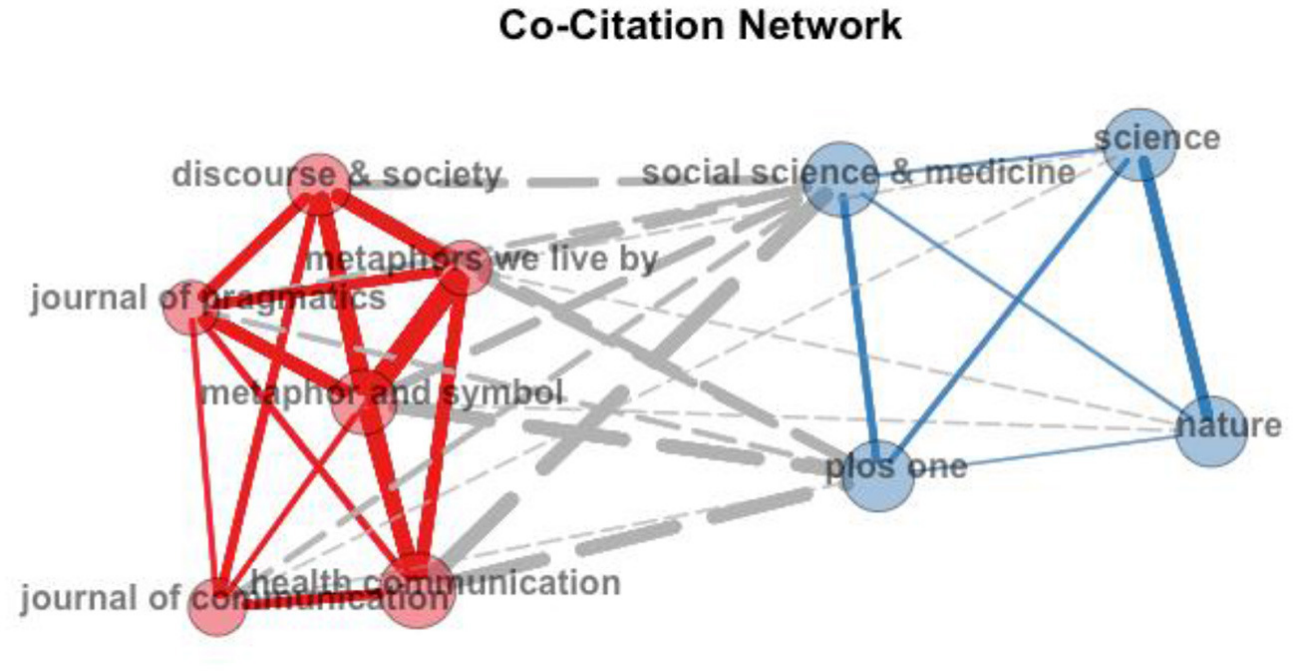
Visualization of the most-co-cited journals.

Eight linguistics and multidisciplinary journals and a seminal book were identified as the most highly co-cited journals, thresholding 15 citations as the lowest number of citations for every source of publication. Journal co-citation is visualized in Figure 5. Two clusters are identified: Cluster 1 represents reputable and traditional linguistics journals such as Discourse and Society, Journal of Pragmatics, and Metaphor and Symbol. The cluster also includes other journals that are familiar to the linguistic field, such as Health Communication, the Journal of Communication, and Johnson and Lakoff's seminal book (red color). Cluster 2 shows a new trend in publishing metaphors in COVID-19 discourse in multidisciplinary journals that are inherently open access, such as PloS One, Nature, Science, or social science journals such as Social Science and Medicine.

#### 4.2.3. Most cited references

According to our findings, five co-citation clusters were identified among the most cited references (Table 4). All cited articles have silhouette values close to 1, indicating a strong thematic relationship between the references and topic of the current investigation.

**Table 4:** Co-citation as per the cited paper.

**Cluster ID**	**Size**	**Silhouette**	**The most relevant cited paper**	**Label (LLR)**	**Average year**
0	28	0.844	Woodgate et al., 2021	Intersubjective experience	2021
1	24	0.85	Benzi and Novarese, 2022	News discourse	2021
2	20	0.772	Gui, 2021	Newspaper coverage	2021
3	20	0.919	Amaireh, 2022	Crisis narrative	2021
4	18	0.768	Semino, 2021	Twitter discourse	2021

Moreover, Table 5 lists the timespan citation of these research articles, burst strength, and sigma value. Sigma value is 1, which indicates the novelty of the research. Three articles and a book were found to have the strongest citation bursts. Details of degree of centrality, sigma values, and citation counts are provided in Appendix 1.

**Table 5:** Timespan citations of the citing references.

**References**	**Burst strength**	**Begin**	**End**	**Σ**
Joye (2010)	2.67	2020	2020	1
Wallis and Nerlich (2005)	1.91	2020	2022	1
Nerlich (2007)	1.43	2021	2022	1
Sontag (1979)	1.43	2021	2022	1

### 4.3. Research themes, trends and hot topics in COVID-19 metaphors

#### 4.3.1. Research themes analysis

**Figure 6:**
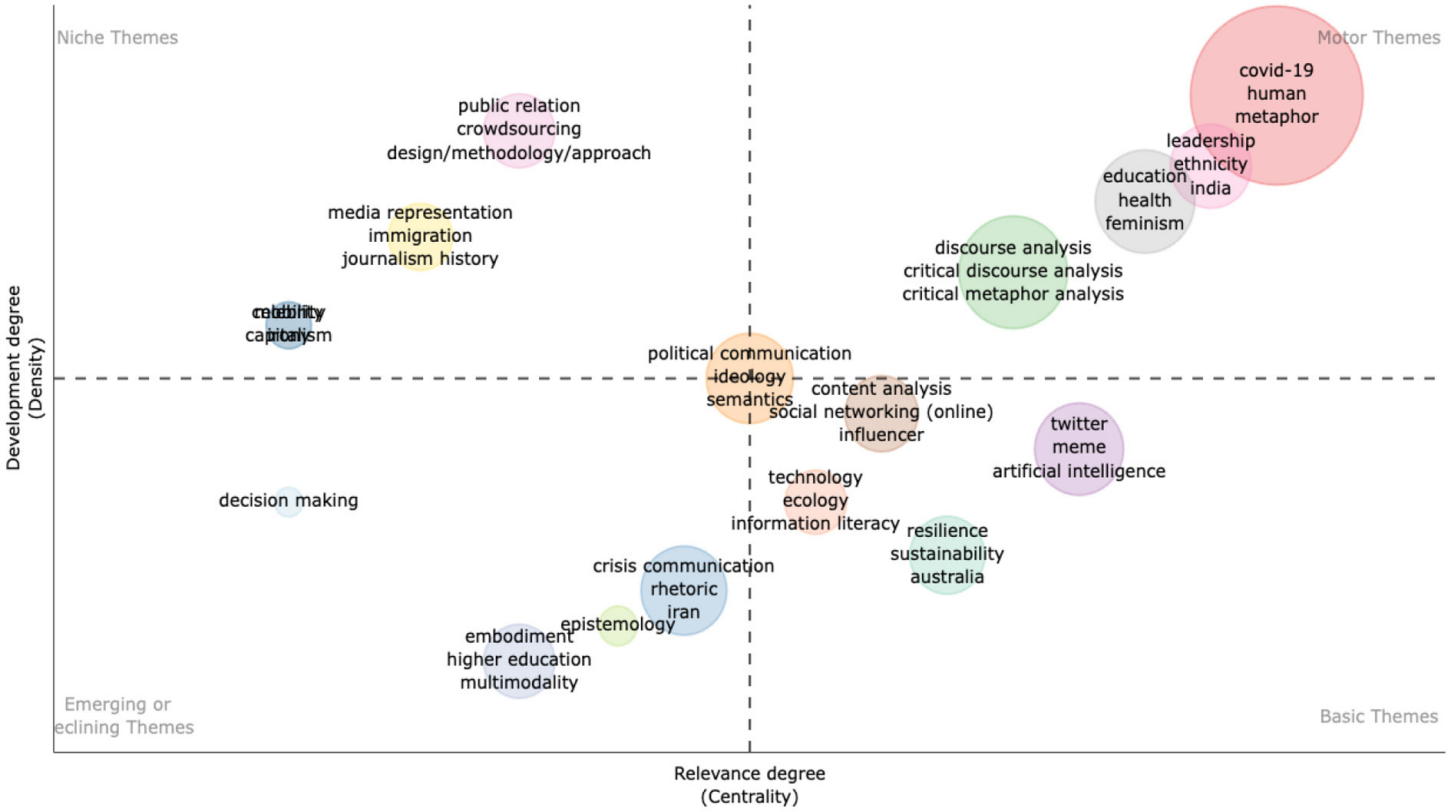
Visualization of thematic mapping.

As shown in Figure 6, the thematic map was constructed based on author keywords and was mapped into four themes: niche (left top), motor (top right), emerging or declining (left bottom), and basic themes (right bottom). In motor themes, research themes that are well-developed are plotted top right and include Cluster 1: COVID-19, human, and metaphor; Cluster 2: discourse, critical discourse, and critical metaphor analyses; Cluster 3: education, health, and feminism; and Cluster 4: leadership, ethnicity, and India. Moreover, there are basic themes in most scholarly production of metaphor research. For example, Cluster 1 includes descriptive research methods such as content analysis, and other keywords such as networking and influencer. The types of social media data are included in Cluster 2 such as twitter, meme, and artificial intelligence. In Cluster 3, basic themes include resilience, sustainability, and Australia. In Cluster 4, basic themes include technology, ecology, and information literacy. In both motor and basic themes, there is a heavy influence of CDA research methods and methodologies in metaphor studies. Niche themes are represented in three clusters (top left): Cluster 1 includes themes such as public relations, crowdsourcing, design/methodology/approach, Cluster 2 includes themes such as media representation, immigration, and journalism history, and Cluster 3 includes celebrities. In emerging or declining themes, Cluster 1 contains themes such as crisis communication, rhetoric, and Iran. In Cluster 2, three themes include embodiment, higher education, and multimodality. Cluster 3 has epistemology as a theme.

#### 4.3.2. Research trends analysis

**Figure 7:**
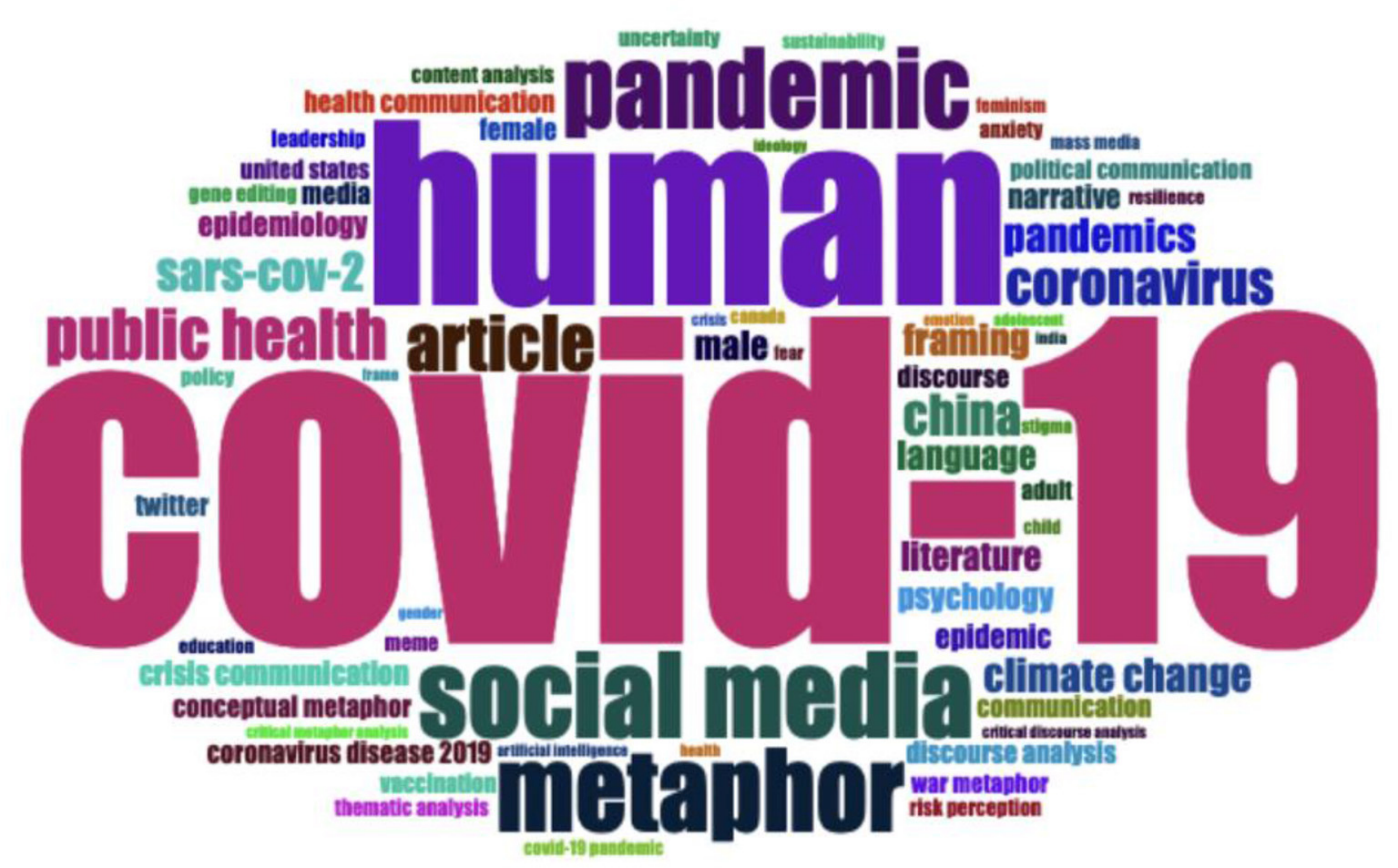
A word cloud of the 65 most frequently used research keywords.

When examining the most frequent research keywords in Figure 7, we noted that the font size correlated positively with the frequency of words; that is, the words used more frequently were seen in bigger font size in the word cloud. The most frequent keywords were related to coronavirus (e.g., COVID-19, coronavirus, SARS-CoV-2, pandemic, and epidemic), metaphor (e.g., metaphor, framing, language, conceptual metaphor, war metaphor, and frame), discourse analysis (e.g., discourse, discourse communication, content analysis, thematic analysis, critical discourse analysis, and critical metaphor analysis), fields of science (e.g., literature, psychology, communication, crisis communication, epidemiology, public health, health communication, political communication, and ideology), and genre of data (e.g., social media, narrative, media, twitter, meme, and mass media).

**Figure 8:**
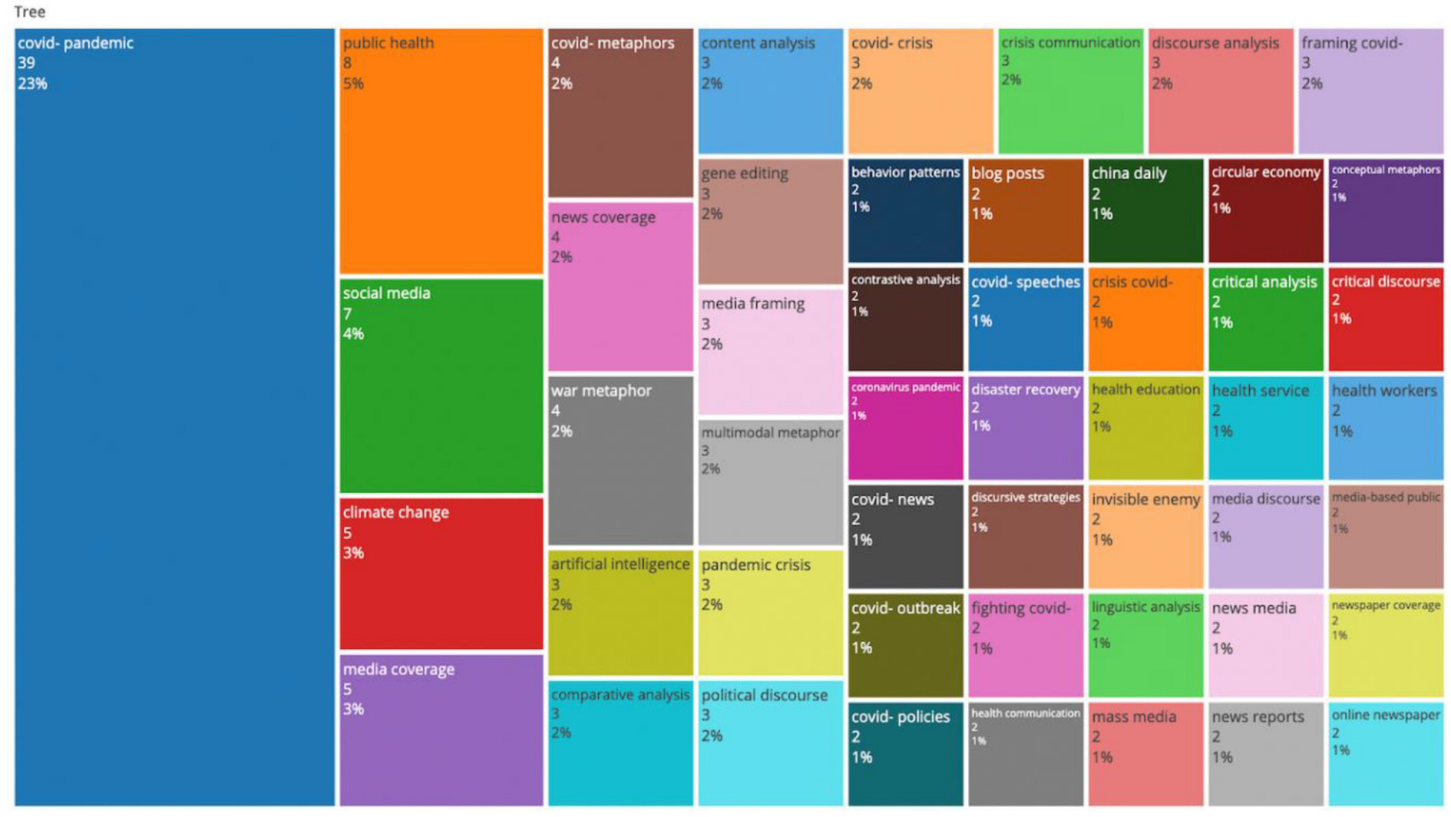
A tree map of the 50 most frequently used bigrams of research titles.

A tree map of the 50 most frequent bigrams of research titles is plotted in Figure 8. Among these research titles, the term “COVID-pandemic” was seen in most published papers (~23% of the documents). The second most published title was public health (8%) followed by social media, climate change, and media coverage (14%). COVID-metaphors, war metaphors, multimodal metaphors, conceptual metaphors, and an invisible enemy composed of 8% of the documents. Other titles were related to methods of analysis (e.g., comparative analysis, content analysis, contrastive analysis, discursive strategies, linguistic analysis, critical analysis, discourse analysis, and critical discourse) and news genres (e.g., blog posts, covid-speeches, China daily, mass media, news reports, newspaper coverage, and online newspapers).

#### 4.3.3. Research hot topics analysis

**Figure 9:**
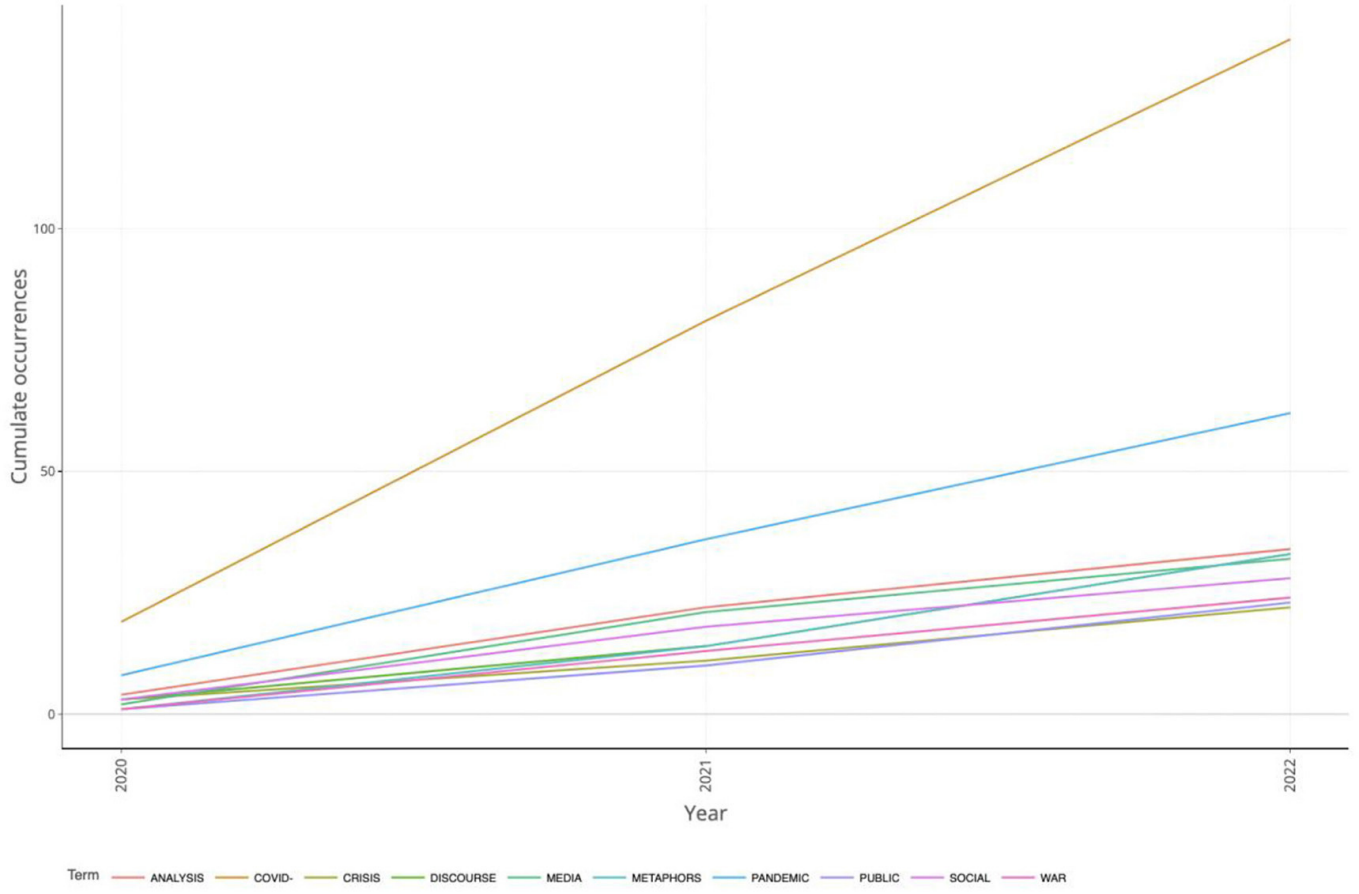
A word dynamic plot of the most frequent unigrams of research titles.

Graphing keyword dynamics helps researchers understand keyword dynamics over time. A graph showing changes in keyword frequency over a period helps choose the best title for a literature review or identify a new research topic. Figure 9 shows an upward trend in author titles from 2020 to 2022. COVID-19 had the highest occurrence during the entire period, and pandemic had the second most frequent occurrence. Analysis, discourse, and war had a similar trajectory of occurrences. Other keywords with similar dynamics included crisis, media, metaphor, public, and social.

## 5. Discussion

As noted by Peng and Hu (2022), Linguistics has made major contributions to the literature on COVID-19, therefore it is worth examining metaphors in COVID discourse. This study provides an overview of research trends in metaphor on COVID-19 discourse by retrieving 327 WoS and Scopus indexed publications. Between 2020 and 2023, the VOSviewer software allowed for the examination of impactful authors, journals, and countries. Additionally, the study examines international cooperation and finds prospective co-authors. Themes, trends, and hot topics analyses deepen researchers' knowledge of trending research subjects and developments.

Research on COVID metaphors has usually increased from 2020 to 2022, and in addition to top-notch metaphor scholars, younger scientists have been interested in the topic. Ahmed Abdel-Raheem was the most prolific author during this period, while George Lakoff and Mark Johnson had the most co-citations. Furthermore, Gema Online Journal of Language Studies has the highest number of COVID-19 metaphors papers. An investigation of international and institutional collaboration to date demonstrates that the United States has played a major role. In the meanwhile, the United Kingdom, Spain, Germany, China, and Netherlands have all contributed significantly to the research topic.

COVID-19 metaphor research is heavily influenced by CDA approaches and methods such as *critical discourse analysis* and *critical metaphor analysis*. Metaphor analysis shows how media use metaphorical language to influence public opinion on controversial issues, the role metaphors play in framing discourse, and the problems associated with using some metaphors to describe COVID-19.

This research has revealed the link between CMT and discourse analysis and has offered additional evidence that research on conceptual metaphors is productive when reviewing data about COVID-19. Recently, we have noticed the emergence of a new research trend that might evolve such as artificial intelligence (AI). Future research trends might focus on AI to analyze patterns of language in different genres and find solutions to guide human behavior during pandemics.

The analysis of COVID metaphors showed that the conceptual metaphor WAR is the most predominant source domain to describe coronavirus and the linguistic metaphor COVID as an invisible enemy is the most frequent linguistic expression used to describe the novel virus. Notwithstanding, most research in metaphor studies has rejected those depictions and looked for alternative conceptualizations for the virus (see Semino, 2021).

Social media and multimodal data offer new and creative data for analyzing metaphors. Multimodal analysis primarily depends on technological developments in data collecting and analysis (Chen et al., 2020), but metaphor analysis remains qualitative in nature. While CDS focuses on evaluating naturally occurring data (Wodak and Meyer, 2009, p. 2), the analysis of memes, for example, provides linguistic evidence that is based on naturally occurring language as opposed to intuitively perceived linguistic occurrences.

However, this study has several limitations. First, the data collection period ended in August 2022, and 4 months of research are not considered. Second, this study only includes English-language Scopus journal articles. In the bibliometric study of metaphor discourse analysis in the pandemic, monographs, collected books, and journal articles in various languages are all relevant. Third, bibliometric analysis entails subjectivity in data collection and analysis (also see Lei and Liu, 2019a; Huan and Guan, 2020). In this study, the interpretation of semantic functions in network visualization might include some subjectivity.

## 6. Conclusion

The COVID-19 epidemic has significantly damaged and disrupted human existence. Linguists have responded to this epidemic by exploring the phenomena of COVID-19 and the impact of language use on human behavior. Metaphor as part of (critical) discourse studies flourished during COVID-19. Therefore, it is crucial to assess its progress and predict its future. This study used bibliometric, network, and thematic mapping to understand COVID metaphor research in discourse and identify recent research foci. Three hundred and twenty-seven publications from January 2020 to August 2022 were retrieved from WoS and Scopus databases. Bibliometric, network, and thematic mapping analyses were employed using Bibliometrix and VOSviewer. The study found that Conceptual metaphor theory made huge contribution in analyzing COVID-19 discourse. The study found also that metaphors that invoked violence framing such as WAR metaphors have been criticized in discourse research. The research production investigated traditional genres (like news) and emerging genres (such as social media and multimodal data), highlighting the role played by metaphors in persuading public discourse.

This study offers a comprehensive overview of metaphor research on COVID-19. Thus, it can serve as a useful springboard for linguists interested in investigating COVID-19 discourses and texts through the lens of leading theories in the field, thereby not only broadening the scope of metaphor research in the pandemic but also generating valuable insights in the fields of pragmatics, metaphor, CDA, and corpus research. These findings may have both theoretical and practical significance for the sub-field of metaphor and discourse.

## Data availability statement

The original contributions presented in the study are included in the article/Supplementary material, further inquiries can be directed to the corresponding author.

## Author contributions

The author confirms being the sole contributor of this work and has approved it for publication.
