# Supplementary material for: Bibliometric, network, and thematic mapping analyses of metaphor and discourse in COVID-19 publications from 2020 to 2022
Source: Front Psychol. 2023 Jan 16;13:1062943. doi: 10.3389/fpsyg.2022.1062943 (PMC9886006; doi:10.3389/fpsyg.2022.1062943)
Supplement: Supplementary file 1 [file Data_Sheet_1.docx]

Supplementary Material

Bibliometric, Network, and Thematic Mapping Analyses of Metaphor and Discourse in COVID-19 Publications from 2020 to 2022

# Supplementary Data

Appendix 1:

| Cluster | Node | | Betweenness | | Closeness | Page Rank |
| --- | --- | --- | --- | --- | --- | --- |
| 1 | | covid-19 | | 127.2590209 | 0.04 | 0.174992223 |
| 1 | | social media | | 0 | 0.023809524 | 0.019612069 |
| 1 | | coronavirus | | 0 | 0.02173913 | 0.014869582 |
| 1 | | china | | 0 | 0.02173913 | 0.012592898 |
| 1 | | climate change | | 0 | 0.02173913 | 0.010316213 |
| 1 | | framing | | 0 | 0.02173913 | 0.012592898 |
| 1 | | epidemic | | 0 | 0.02173913 | 0.011834003 |
| 1 | | communication | | 0 | 0.024390244 | 0.016844079 |
| 1 | | health communication | | 0 | 0.02173913 | 0.011075108 |
| 2 | | human | | 61.74051236 | 0.035714286 | 0.139816241 |
| 2 | | article | | 8.857738863 | 0.03030303 | 0.070750148 |
| 2 | | public health | | 0 | 0.024390244 | 0.0205814 |
| 2 | | language | | 0 | 0.020408163 | 0.009841398 |
| 2 | | male | | 0.263157895 | 0.02173913 | 0.027673237 |
| 2 | | psychology | | 1.081772112 | 0.026315789 | 0.031611886 |
| 2 | | coronavirus disease 2019 | | 0 | 0.026315789 | 0.03729418 |
| 2 | | female | | 0 | 0.021276596 | 0.021524418 |
| 3 | | metaphor | | 0 | 0.027027027 | 0.05183774 |
| 3 | | pandemic | | 3.870490245 | 0.03030303 | 0.083035927 |
| 3 | | sars-cov-2 | | 0.479234503 | 0.028571429 | 0.062873099 |
| 3 | | pandemics | | 1.303613282 | 0.029411765 | 0.066964653 |
| 3 | | literature | | 0.144459813 | 0.027777778 | 0.049385596 |
| 3 | | epidemiology | | 0 | 0.027027027 | 0.042081003 |
|  | |  | |  |  |  |
